# Erythritol-enriched powder and oral biofilm regrowth on dental implants: an *in vitro* study

**DOI:** 10.4317/medoral.24622

**Published:** 2021-03-27

**Authors:** Patricia Amate-Fernández, Rui Figueiredo, Vanessa Blanc, Gerard Àlvarez, Rubén León, Eduard Valmaseda-Castellón

**Affiliations:** 1 DDS, PhD student. Faculty of Medicine and Health Sciences of the University of Barcelona, Spain; 2 DDS, MS, PhD. Tenured lecturer. Oral Surgery and Implantology Department, Faculty of Medicine and Health Sciences of the University of Barcelona, Spain; 3 Researcher at the IDIBELL Institute, Barcelona, Spain; 4 MS, PhD. Microbiology Department Director. Dentaid Research Center, Cerdanyola del Vallès, Barcelona, Spain; 5 MS, PhD. Microbiology Department Researcher. Dentaid Research Center, Cerdanyola del Vallès, Barcelona, Spain; 6 MS, PhD. R&D Manager. Dentaid Research Center, Cerdanyola del Vallès, Barcelona, Spain

## Abstract

**Background:**

Peri-implant mucositis and peri-implantitis are the main biological complications associated with dental implants. Since most authors agree that bacteria play a major etiological role, the main aims of this study were to determine if a formulation of erythritol and chlorhexidine applied with an air polishing system inhibits biofilm regrowth over dental implants and to compare the decontamination capacity of this therapy with that of mechanical removal by saline and gauze.

**Material and Methods:**

A multispecies biofilm (P. *gingivalis*, *A. actinomycetemcomitans*, *F. nucleatum*, *A. naeslundii*, *V. parvula* and *S. oralis*) was grown for 14 days on 52 dental implants in an artificial mouth. These implants were divided into three groups according to the applied treatment: 14 negative control (CON), 19 erythritol-chlorhexidine (ERY) and 19 gauze with saline (GAU) samples. Twelve dental implants from the ERY and GAU groups and 8 implants from the CON group were re-incubated for 7 additional days after treatment. The bacterial count was performed by quantitative polymerase chain reaction (qPCR) using propidium monoazide (PMA). A descriptive and bivariate analysis of the data was performed.

**Results:**

The erythritol and chlorhexidine formulation significantly inhibited biofilm regrowth in comparison with the mechanical treatment (GAU), since a significant decrease in all the species was observed in the ERY group (except for *Aggregatibacter actinomycetemcomitans*). The antibiofilm and antibacterial capacity of the two active treatment groups (ERY and GAU) was similar for a 14 days multispecies *in vitro* biofilm, except for the lower count of *A. naeslundii* in the GAU group.

**Conclusions:**

The use of erythritol powder with chlorhexidine applied with an air polishing system reduces biofilm regrowth over dental implants when compared with mechanical removal by saline and gauze. This effect might be beneficial for patients included in peri-implant maintenance programs.

** Key words:**Dental implants, biofilms, peri-implantitis, erythritol, chlorhexidine.

## Introduction

Dental implants have become the gold standard when aiming at reconstruction of missing teeth. Several studies have proven dental implants to be a reliable alternative for providing function and aesthetics with long-term success. However, with increasing numbers of fixtures being installed yearly, there has also been a significant increase in peri-implant diseases (1).

Mucositis and peri-implantitis are the main biological complications associated with dental implants (2,3). Data indicate that bacteria play a major etiological role in the development of these complications, several treatment strategies have been developed with the aim of reducing the bacterial count, decontaminating the implant surface and removing the biofilm (4). Biofilms are surface-adhered microbial communities embedded in a self-produced matrix (5). These organized communities represent a significant health risk due to their resistance to host-defense mechanisms and their decreased susceptibility to conventional antimicrobials. Biofilm-mediated resistance has been attributed to impaired penetration of antimicrobials through the matrix, increased expression of drug-resistance genes, and reduced metabolic activity of cells residing in the biofilm. Because of their involvement in bacterial infections in humans, biofilms have been the subject of intensive research for many years (5).

 Polymerase chain reaction (PCR) techniques have shown several advantages over traditional microbiologic culture methods. They are faster and have a higher specificity and sensitivity for identifying and quantifying oral bacteria. Nevertheless, PCR might overestimate the number of active bacteria because it does not differentiate DNA coming from live or dead microorganisms. Thus, the use of propidium monoazide (PMA) is of great interest since it allows to detect cell membrane integrity, distinguishing viable and irreversibly damaged cells (6-10).

Regarding the treatment of peri-implant diseases, a variety of different approaches have been proposed, ranging from non-surgical therapy to laser disinfection or to surgical treatments with either resective or regenerative approaches (11). The ideal treatment should focus not only on removing pathogens from the implant surfaces, but also on preventing bacterial regrowth over the area. This is a crucial issue, since one of the main treatment goals should be to prevent the adhesion of primary colonizers to the recently decontaminated implant surfaces. However, knowledge on this process is still very scarce.

A minimally abrasive powder containing erythritol and 3% chlorhexidine has been considered a promising treatment option for the removal of subgingival biofilm (12,13). A recent systematic review and meta-analysis showed that erythritol/chlorhexidine was superior or equal to other methods when applied over contaminated implant surfaces, and was more biocompatible (14). However, few studies have been performed to evaluate the anti-biofilm regrowth properties of this formulation.

For this reason, the present *in vitro* study was conducted with the following aims: to determine if a formulation of erythritol and 3% chlorhexidine applied with an air polishing system inhibits biofilm regrowth over dental implants and to compare the decontamination capacity of this therapy with those of mechanical removal with saline and gauze.

## Material and Methods

A randomized, single-blind, *in vitro* study was carried out. Fifty-two dental implants (Avinent, Santpedor, Barcelona, Spain) were subjected to *in vitro* multi-species biofilm formation in the microbiology department of the Dentaid Research Center (Dentaid SL, Cerdanyola del Vallès, Spain). Decontamination of the implants was randomly performed either with erythritol powder with 3% of chlorhexidine (Air Flow Powder Plus, EMS, Nyon, Switzerland) using an air-flow device (EMS, Nyon, Switzerland) or with a gauze swab with sterile saline solution. A single clinician performed these procedures for the time considered necessary to remove the biofilm adequately according to the clinician’s professional criterion. The implants were then coded to avoid bias in the microbiology analysis.

- Biofilm development on implant surfaces

Oral biofilms were grown in an artificial mouth model in an anaerobic atmosphere (15) using the following bacterial species: *Streptococcus oralis* DSM 20627, *Veillonella parvula* NCTC 11810, *Actinomyces* naeslundii DSM 17233, *Fusobacterium nucleatum* DSM 20482, *Porphyromonas *gingivalis** ATCC 33277 and *Aggregatibacter actinomycetemcomitans* DSMZ 8324. The bacteria were kept on blood agar plates (Oxoid No. 2; Oxoid Ltd, Basingstoke, UK) with 5% horse blood, 5mg/L hemin and 1 mg/L menadione at 37ºC under anaerobic conditions, while BHI-II medium was employed for both the liquid cultures and biofilm growth. All the species were inoculated simultaneously from exponential phase cultures, except for *P. *gingivalis**, which was inoculated 24 hours previously in the bioreactor. The bacteria were kept in a continuous culture for 5 days. Subsequently, they were transferred to the device where the implants were located, bathing them totally for 14 days at a flow rate of 30 mL/h, in anaerobic conditions, at 37ºC and pH 7.2 (15).

- Experimental treatments

**Figure 1:**
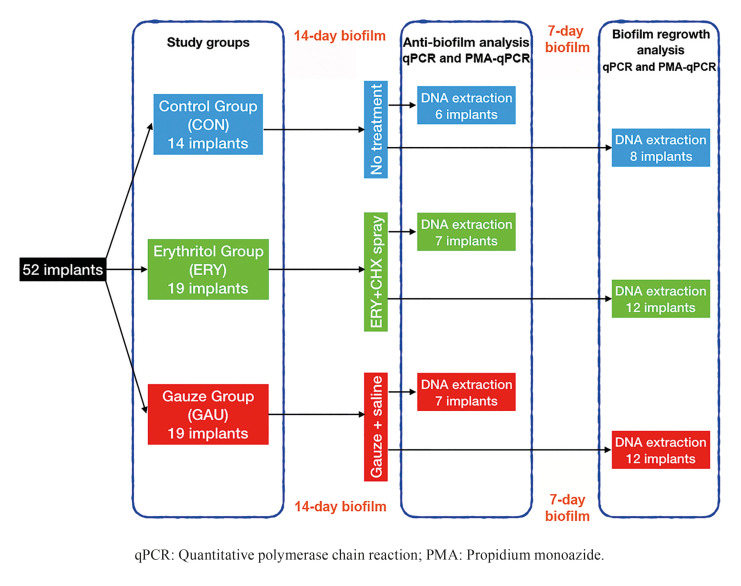
Flow diagram of the present study.

After 14 days in the artificial mouth, the implants were removed from the device and assigned randomly, using a computer-generated sequence (www.randomization.com, accessed on February 18, 2019), to one of the study groups: erythritol powder with 3% of chlorhexidine (ERY group); gauze with sterile saline (GAU group) or negative control (CON group) (Fig. 1). Fourteen negative control implants were immersed three times in phosphate buffered saline (PBS) (137 mM NaCl, 2.7 mM KCl, 10 mM Na2HPO4, 2 mM KH2PO4, pH 7.4). Of these, 6 were then processed for genomic DNA extraction and 8 were placed in a new sterile vessel for regrowth. In the ERY group, 19 implants were treated with chlorhexidine-enriched erythritol powder, applied with an air-polishing device. Seven were processed for genomic DNA extraction and 12 were re-incubated for 7 days after treatment. In the GAU group, the same number of implants were treated mechanically (gauze with sterile saline) and analyzed (Fig. 1) or used for regrowth.

The regrowth process took place under the same conditions as the initial biofilm development. After this incubation time, all the regrowth implants were processed for genomic DNA extraction.

-Propidium monoazide (PMA) treatment, DNA extraction and quantitatve PCR (qPCR)

qPCR was used to determine the total number of cells of each species that formed the biofilms on the implants, while the live cell count was determined by PMA-qPCR (16). For this purpose, the implants were washed by immersing them three times in PBS. The biofilms were dispersed using a vortex for 5 minutes in 1 mL of PBS. The PMA treatment prior to genomic DNA extraction was performed as described by Àlvarez *et al*. (16). Genomic DNA extraction was effected using the QIAamp DNA Mini Kit (Qiagen, Hilden, Germany), following a previously published protocol (15). Quantitative PCR was performed with a LightCycler 480 II Instrument and the LightCycler 480 II Probes Master kit (Roche Diagnostics, Penzberg, Germany) and specific primers (Invitrogen Life Technologies, Carlsbad, CA, USA) and probes (Applied Biosystems, UK and Roche Diagnostics, Penzberg, Germany) were used. Data analysis was performed with LightCycler 480 Software 1.5 (Roche Diagnostics) using the second derivative maxim method. The qPCR reaction was conducted using an initial cycle of 95°C for 10 minutes, followed by 40 cycles of denaturation at 95°C for 10 seconds, annealing at 60 °C for 30 seconds, and extension at 72°C for 1 second. Standard curves for each bacterial species were developed as described in Àlvarez *et al*. (16).

- Microscopic analysis

Two implants from each group were randomly selected for analysis by confocal laser scanning microscopy (Leica TCS SP5, Leica Microsystems, Heidelberg, Germany). After a washing procedure to remove non-adhered bacteria, the implant biomass was dyed with SYTO9 nucleic acid stain (Molecular Probes, Eugene, OR, USA) at room temperature in the dark for 10 minutes. Six fields per implant were acquired with a 10x objective, with the white laser set at 482 nm. Imaris® v.7.1 software (Bitplane AG, Badenerstrasse, Zurich, Switzerland) was used to obtain a 3D reconstruction of each field from the optical sections. Due to the results found in the ERY group (recolonization), 2 negative control implants and 2 implants treated with erythritol were examined under a scanning electron microscope (Merlin FE-SEM®, Carl Zeiss, Oberkochen, Germany) at 30,000x in order to detect surface alterations after treatment.

- Statistical analysis

A descriptive analysis of the data was performed with the Statistical Package for Social Sciences software (SPSS v22.0; IBM Corp, Armonk, New York). Kruskal Wallis tests and Mann-Whitney U-tests were employed to detect differences between the 3 groups. The level of significance was set at *p* < 0.05.

## Results

The results concerning the effect of the 3 treatments on a 14-day multispecies biofilm can be observed in Table 1. The groups showed similar qPCR results for most of the bacteria. However, the mechanical treatment (GAU group) had a lower total count (qPCR) of *A. naeslundii* and *P. *gingivalis** than the CON group (*p*= 0.01 and *p*= 0.026, respectively). No significant differences were found when comparing the two active treatment groups (ERY and GAU), except in *A. naeslundii*, which had a lower bacterial count in the GAU group (*p*= 0.022; Table 1). Regarding the PMA-qPCR results, the number of live *P. *gingivalis** cells was significantly lower in the ERY group than in the CON group, while the number of live *A. actinomycetemcomitans* cells was lower in both treatment groups (ERY and GAU) than in the CON group.

**Figure 2:**
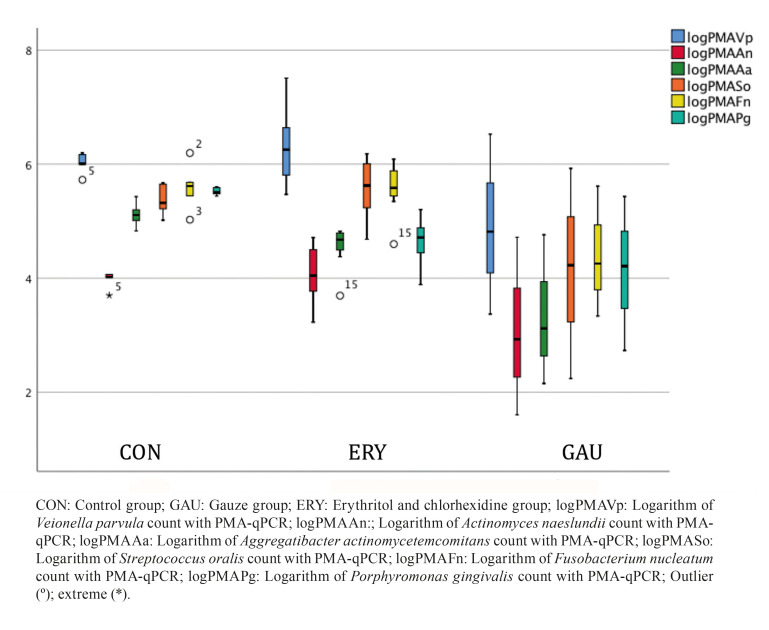
Comparison between the PMA-qPCR ratios of the 3 groups following treatment of a 14-day biofilm.

**Figure 3:**
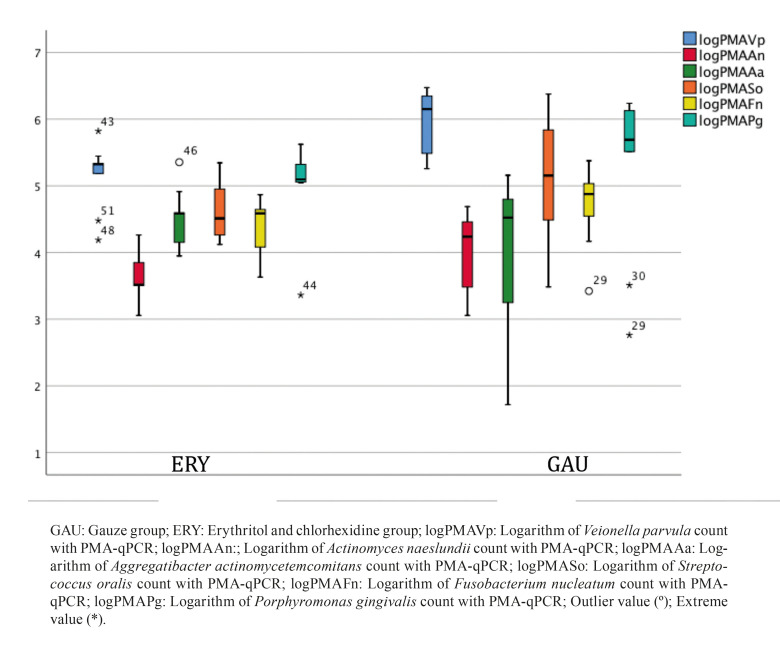
Comparison between the PMA-qPCR ratios of the 2 active groups after therapy and 7-day biofilm regrowth.

The outcomes of the second phase of the study (biofilm regrowth after treatment), which are related with the main study aim, are presented in Table 2. In comparison with the standard mechanical therapy (GAU), the ERY group was found to present a significant reduction in biofilm regrowth after therapy for all species except *A. actinomycetemcomitans* (qPCR and PMA-qPCR) and *S. oralis* (PMA-qPCR). To evaluate the resilience of the biofilms after application of the therapies, the PMA-qPCR ratios (following treatment after 14 days of biofilm growth and after 7 days of biofilm regrowth) were compared (Fig. 2, Fig. 3).

**Table 1:**
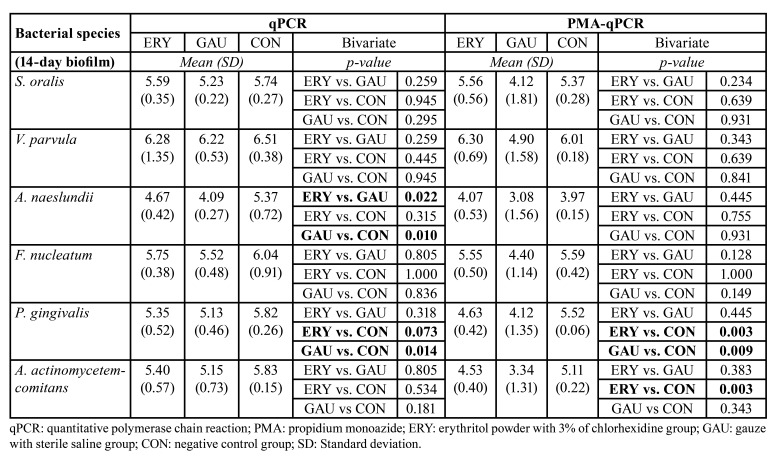
Main results for the 3 treatment groups (ERY, GAU and CON) in the first phase of the study (after treatment following 14 days of biofilm growth) stratified by logarithms of bacterial species.

**Table 2:**
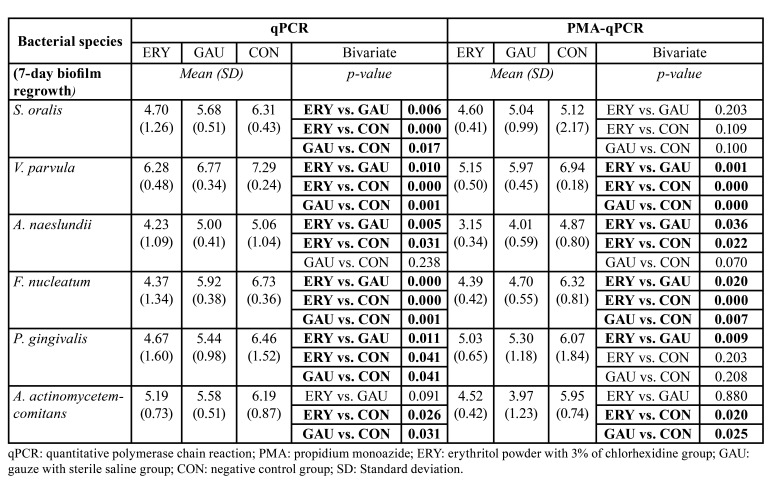
Main results for the 3 treatment groups (ERY, GAU and CON) in the second phase of the study (after 7 days of biofilm regrowth following treatment), stratified by logarithms of bacterial species.

**Figure 4:**
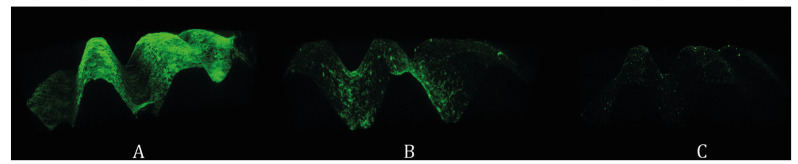
Confocal microscopy images after 7 days of biofilm recolonization of the surface of a dental implant. A: Control group, B: Gauze group, C: Erythritol and chlorhexidine group.

The ERY biofilms showed the lowest ratios for most of the species, indicating low regeneration of the biofilm after this treatment. The confocal optical microscope images also support these results, since the biofilm coverage area was smaller on the ERY group implants (Fig. 4).

Scanning electronic microscopy (SEM) disclosed that the 2 implants of the ERY group displayed attached particles of approximately 0.1 m in size firmly attached to the implant surface. These particles were not found in the negative control implants.

## Discussion

The present study shows that applying an erythritol-enriched powder with 3% of chlorhexidine through an air-flow device inhibits biofilm regrowth on dental implants in comparison with debridement with a gauze with sterile saline solution. However, these therapies have proved to give similar results for removing already established biofilms.

This research has some limitations that must be mentioned. Firstly, the *in vitro* biofilm employed is not exactly the same as the one that a clinician might find in a patient. However, this oral biofilm included several bacterial species (multispecies) and was produced using a dynamic model (artificial mouth) that simulated real crevicular flow conditions. Thus, this method overcomes most of the limitations of the static *in vitro* biofilm traditionally employed (15,17). Moreover, using PMA-qPCR provides important information, since it determines the bacterial count of live microorganisms present in the biofilm. Secondly, the therapies were applied under ideal conditions of accessibility and light. Certainly, this allowed the researcher to perform a more thorough decontamination of the implants in comparison with the standard clinical environment. For example, the lower areas of the implants’ threads are usually more difficult to access with air-polishing systems, since a bone defect might prevent direct application of the powder in these zones (18). Nevertheless, this situation probably affected both active treatment groups equally and therefore did not favor one group over the other. Finally, some available antibiofilm treatments could not be included in the present study. Thus, future research should compare the disinfection properties of the erythritol-chlorhexidine formulation with those of other active therapies (Er:YAG laser, chlorhexidine solutions, glycine powder, among others).

Peri-implant diseases are the most prevalent biological complication associated with dental implants. The estimated prevalence of peri-implantitis ranges from 14% to 30%, while mucositis affects 32% to 54% of patients (19). A recently-published private-practice sample with full-arch rehabilitations has shown that these Figures might be even higher, as more than half of the patients were diagnosed with a peri-implant disease (20). According to the 2017 World Workshop on the Classification of Periodontal and Peri-Implant Diseases and Conditions (21), peri-implantitis is a plaque-associated pathological condition occurring in tissues around dental implants, characterized by inflammation in the peri-implant mucosa and subsequent progressive loss of supporting bone. Initial bacterial colonization at implant surfaces occurs within 30 minutes, while a mature subgingival microbiota can be observed within a week (22). According to Cortés-Acha *et al*. (23), a wide variety of bacteria can be identified in healthy dental implants after 14 days of exposure to the oral environment. Also, a global plaque coverage of the implants is expected, even in the subgingival area (24). All this information highlights that plaque control and biofilm removal are paramount for preventing and treating these biological complications. Chemical and physical (mechanical or laser) decontamination strategies have been described in the literature (4). A meta-analysis to determine the most effective non-surgical therapy for the management of peri-implantitis concluded that local application of antibiotics, the use of glycine-powder applied with an air-polishing system, and Er:YAG laser seemed to significantly reduce soft tissue inflammation (bleeding on probing: BoP) (25). However, to the best of the present authors’ knowledge there is no clear evidence that any mechanical or chemical biofilm decontamination technique is superior to others. This also seemed to be the case in the present sample, where the standard mechanical therapy (gauze with sterile saline) had a similar antibacterial effect to that of the erythritol-chlorhexidine treatment. However, this paper adds new and clinically relevant information regarding bacterial recolonization of a recently decontaminated dental implant surface. The selection of the most suiTable decontamination therapy should take into consideration not only the immediate anti-biofilm effect but also its duration. Indeed, inhibition of bacterial regrowth after therapy might play a critical role in the prevention and progression of both mucositis and peri-implantitis. According to the results of the present study, erythritol-enriched powder with 3% of chlorhexidine seems to be a valid option for use in peri-implant maintenance programs, since it might inhibit colonization of the dental implants during the first days after treatment.

Air-polishing systems have been used in dentistry for several indications, some of which seek the removal of oral biofilms (26,27). Some *in vitro* studies have shown that air-polishing sprays seem to achieve better biofilm removal outcomes than other mechanical systems (e.g., plastic curettes and vector system) or lasers (Er:YAG and Er,Cr:YSGG) on micro-structured titanium surfaces (26). However, abrasive substances like sodium bicarbonate might cause undesirable alterations to implant surfaces (28-30). Glycine powder grants the same antibiofilm efficacy with minimal damage to the titanium, due to its low abrasiveness (29). Other agents that have also been tested include erythritol, which produces DNA and RNA depletion, attenuates extracellular matrix production, and alters dipeptide acquisition and amino acid metabolism, leading to inhibition of biofilm development (31). Erythritol has shown promising results under *in vitro* conditions, but also in real clinical scenarios. A recent randomized clinical trial has demonstrated the effectiveness of this powder in removing dental plaque during repeated instrumentation of residual pockets in supportive periodontal therapy (12). To summarize, erythritol seems to induce a change in the oral microbiota, shifting it towards a more favorable environment where the traditional colonizers are predominant (32). One possible explanation for these positive outcomes might be related to the attachment of erythritol/chlorhexidine particles to the dental implants. Indeed, implants from the ERY had 0.1 m particles attached to the implant surface. More studies are required to confirm that these particles have a clinically relevant effect.

In conclusion, the use of erythritol powder with 3% chlorhexidine applied with an air polishing system seems to inhibit biofilm regrowth over recently decontaminated dental implants. However, this combination of erythritol/chlorhexidine seems to have a similar decontamination capacity of a simple mechanical removal (gauze with saline) in already established oral biofilms. Further studies are required to compare this formulation with other decontaminants and to validate the results of this *in vitro* study.
